# Pulsed field atrial fibrillation ablation with variable loop circular catheter: outcomes from large US registry

**DOI:** 10.1093/europace/euag226

**Published:** 2026-08-26

**Authors:** Fengwei Zou, Justin Song, Karen Lee, Nikil Prasad, Sarah Xu, Ariel Gidon, Shubhi Pandey, Jishu Motta, Lowell Safren, Cristian Zamora Rosales, Yuval Shafir, Jose Matias, Nils Guttenplan, Dhanunjaya Lakkireddy, Vincenzo Mirco La Fazia, Sanghamitra Mohanty, Andrea Natale, Xiaodong Zhang, Luigi Di Biase

**Affiliations:** Montefiore-Einstein Center for Heart and Vascular Care, Montefiore Medical Center, Albert Einstein College of Medicine, 111 E 210th street, Bronx, NY 10467, USA; Montefiore-Einstein Center for Heart and Vascular Care, Montefiore Medical Center, Albert Einstein College of Medicine, 111 E 210th street, Bronx, NY 10467, USA; Montefiore-Einstein Center for Heart and Vascular Care, Montefiore Medical Center, Albert Einstein College of Medicine, 111 E 210th street, Bronx, NY 10467, USA; Montefiore-Einstein Center for Heart and Vascular Care, Montefiore Medical Center, Albert Einstein College of Medicine, 111 E 210th street, Bronx, NY 10467, USA; Montefiore-Einstein Center for Heart and Vascular Care, Montefiore Medical Center, Albert Einstein College of Medicine, 111 E 210th street, Bronx, NY 10467, USA; Montefiore-Einstein Center for Heart and Vascular Care, Montefiore Medical Center, Albert Einstein College of Medicine, 111 E 210th street, Bronx, NY 10467, USA; Montefiore-Einstein Center for Heart and Vascular Care, Montefiore Medical Center, Albert Einstein College of Medicine, 111 E 210th street, Bronx, NY 10467, USA; Montefiore-Einstein Center for Heart and Vascular Care, Montefiore Medical Center, Albert Einstein College of Medicine, 111 E 210th street, Bronx, NY 10467, USA; Montefiore-Einstein Center for Heart and Vascular Care, Montefiore Medical Center, Albert Einstein College of Medicine, 111 E 210th street, Bronx, NY 10467, USA; Montefiore-Einstein Center for Heart and Vascular Care, Montefiore Medical Center, Albert Einstein College of Medicine, 111 E 210th street, Bronx, NY 10467, USA; Montefiore-Einstein Center for Heart and Vascular Care, Montefiore Medical Center, Albert Einstein College of Medicine, 111 E 210th street, Bronx, NY 10467, USA; Montefiore-Einstein Center for Heart and Vascular Care, Montefiore Medical Center, Albert Einstein College of Medicine, 111 E 210th street, Bronx, NY 10467, USA; Montefiore-Einstein Center for Heart and Vascular Care, Montefiore Medical Center, Albert Einstein College of Medicine, 111 E 210th street, Bronx, NY 10467, USA; Kansas City Heart Rhythm Institute, University of Missouri-Columbia, Overland Park, KS, USA; Texas Cardiac Arrhythmia Institute, St David's Medical Center, Austin, TX, USA; Texas Cardiac Arrhythmia Institute, St David's Medical Center, Austin, TX, USA; Texas Cardiac Arrhythmia Institute, St David's Medical Center, Austin, TX, USA; Montefiore-Einstein Center for Heart and Vascular Care, Montefiore Medical Center, Albert Einstein College of Medicine, 111 E 210th street, Bronx, NY 10467, USA; Montefiore-Einstein Center for Heart and Vascular Care, Montefiore Medical Center, Albert Einstein College of Medicine, 111 E 210th street, Bronx, NY 10467, USA

**Keywords:** Pulsed field ablation, Atrial fibrillation catheter ablation, Electroporation, Variable loop circular catheter, VARIPULSE

Pulsed field ablation (PFA) is becoming a mainstream energy choice in atrial fibrillation (AF) catheter ablation.^1–4^ The VARIPULSE platform [variable loop circular catheter (VLCC), TRUPULSE generator, and CARTO 3 mapping system, Biosense Webster, Inc., Irvine, CA, USA; a Johnson & Johnson MedTech company] is a safe and efficacious system to perform pulmonary vein isolation (PVI). Real-world outcomes for VLCC AF ablation are limited after regulatory approved 30 mL/min irrigation rate. Recent results from the VARIPURE registry demonstrated good long-term success and safety in European patients.^5^ Long-term follow-up evidence for VLCC is lacking in the US population. We aim to report the real-world safety, efficacy and follow-up outcomes of VLCC AF ablation from a large high-volume centre US registry.

The cardiac electrophysiology research registry at Montefiore Medical Center tracks safety and performance of electrophysiology procedures performed in the institution. All patients underwent AF ablation or concomitant AF ablation and left atrial appendage occlusion (LAAO) using VLCC were screened from the registry. Baseline demographic information, comorbidities, medication use, echocardiographic parameters and procedure-related information including skin-to-skin procedure time, fluoroscopy time, ablation lesion sets, number of PF lesions, complications, and acute success were summarized at the time of procedure. Acute procedural success is defined as exit/entrance block of targeted PVs and absence of any electrical potentials in non-PV triggers at end of procedure. Differential pacing is performed to confirm linear blocks. Posterior wall isolation (PWI) is performed by positioning the VLCC at the centre of the PW and delivering 9 overlapping lesions across the PW, extending superiorly to the roof, inferiorly to the level of the lower PVs, and laterally to overlap the PV antra while ensuring adequate contact.^6^ Superior vena cava (SVC) isolation is performed by placing the VLCC at the level of pulmonary artery under intracardiac echocardiography (ICE) and applying two PF lesions with rotation. All patients received routine follow-up within 2 months, at 6 months, 12 months and yearly after the procedure. Continuous rhythm monitors were prescribed at the provider’s discretion and were reviewed at the following visit. ECGs were performed in all clinic visits. All non-routine follow-ups were also individually reviewed and rhythm was assessed. Freedom from AF/AFL recurrence (>30 s) were evaluated with/without antiarrhythmic medications (AAD) and a 60-day blanking period was utilized. To assess safety relate to the VLCC catheter, acute and follow-up events were analysed for the ablation only cohort. This data analysis is approved by the institutional review board.

From October 22 2024 to April 9 2026, 501 patients (43.5% female, mean age 66.5 ± 10.7 years) underwent AF ablation alone (393 patients) or concomitant ablation and LAAO (108 patients) using VLCC. AF subtypes included 246 (49.1%) paroxysmal and 255 (50.9%) non-paroxysmal. Patients in the non-paroxysmal group had higher prevalence of congestive heart failure (49.2% vs. 24.8%), chronic kidney disease/end stage renal disease (22% vs. 12.2%), higher BMI (32.1 ± 7.4 vs. 30.1 ± 5.7), lower LVEF (50.9 ± 13.7% vs. 57 ± 11.1%), larger LA size (4.4 ± 0.7 cm vs. 3.9 ± 0.7), and higher CHA_2_DS_2_-VAS_C_ score (3.5 ± 1.6 vs. 3.1 ± 1.7) (*Figure 1A*). Higher irrigation rate during pulse delivery was used in 467 (93.2%) cases and all cases were performed under general anaesthesia and ICE. All patients underwent PVI unless PVs were mapped to be durably isolated from previous ablations. Additional PFA lesions included PWI (339, 67.7%) and SVC isolation (250, 49.9%). The mean number of PFA applications was 27.5 ± 10.8. Radiofrequency energy was used to perform cavotricuspid isthmus and mitral isthmus linear ablations. Median total procedure time for the entire cohort was 76.0 min (IQR 63.0–96.0 min). Median procedure time for ablation alone was 73.0 min (IQR 60.0–95 min) vs. 79.0 min (IQR 71.0–96.0 min) for concomitant ablation and LAAO. Median fluoroscopy time was 10.1 min (IQR 4.8–15.6 min) for the entire cohort and 96 (19.2%) procedures were performed without fluoroscopy. Acute success was achieved in all patients. To assess catheter-related safety, patients receiving AF ablation alone had acute complication rate of 0.8% (3/393), including two vascular complications not requiring intervention or prolonged hospital stay and one transient ST elevation from air embolism during irrigation exchange without later clinical impact. While higher catheter irrigation rate has been associated with better safety profile, none of the complications were related to irrigation rate.^7^ No late complications were noted during follow-up.

**Figure 1:**
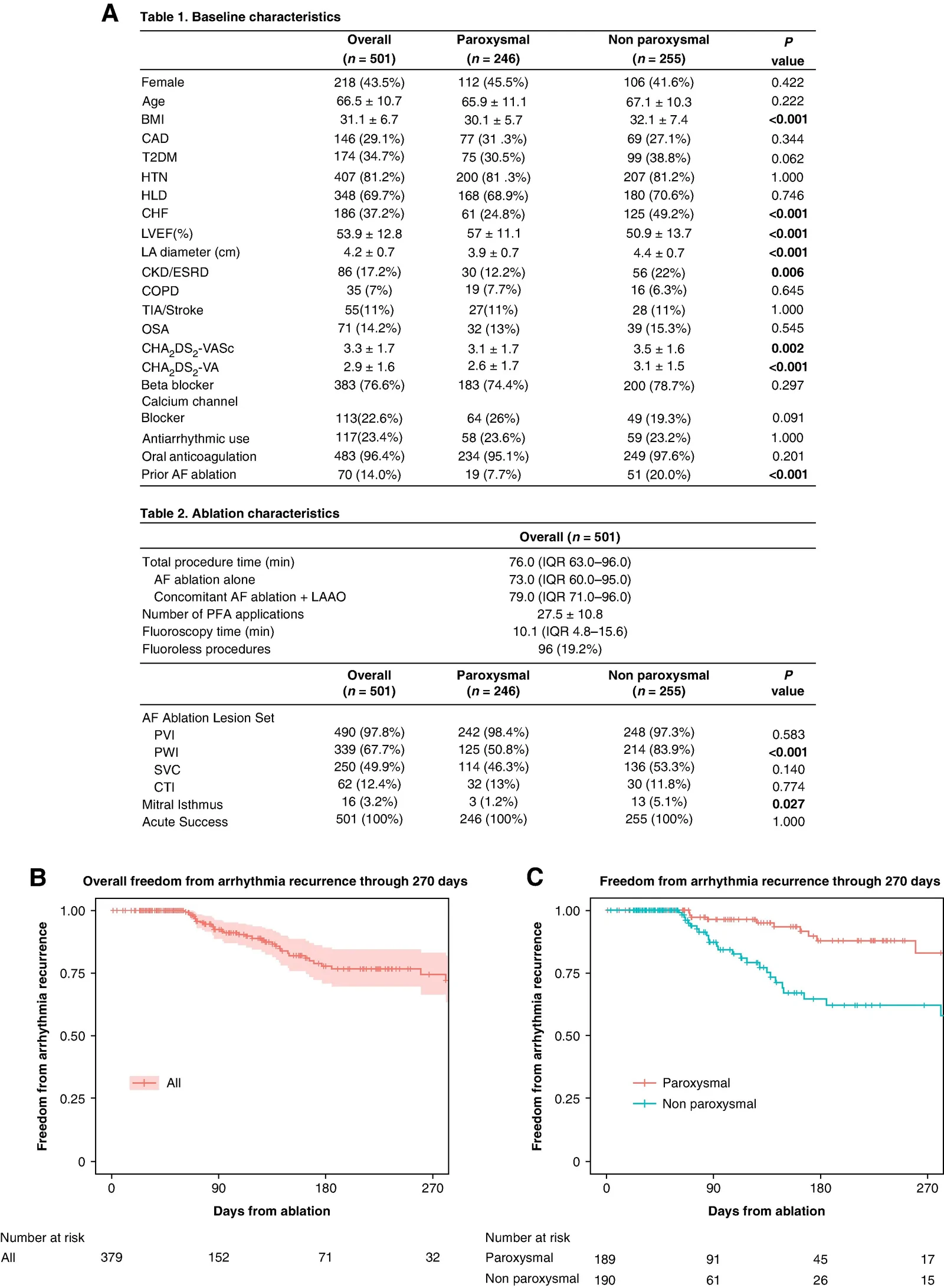
Baseline and ablation characteristics and freedom from atrial arrhythmias. (*A*) Table 1 shows baseline characteristics of the cohort categorized by paroxysmal vs. non- paroxysmal AF. Patients with non-paroxysmal AF has higher BMI, more CHF, larger LA, lower LVEF and higher CHA_2_DS_2_-VASc score. Table 2 shows ablation characteristics including procedure time, number of PFA lesions, fluoroscopy time and lesion set performed. (*B*) and (*C*) Kaplan-Meier survival curve for freedom of atrial arrhythmias for the overall cohort and categorized by paroxysmal vs. non-paroxysmal AF. AF, atrial fibrillation; BMI, body mass index; CAD, coronary artery disease; CHA_2_DS_2_-VA, congestive heart failure, hypertension, age ≥75 years, diabetes mellitus, prior stroke or transient ischaemic attack, vascular disease, and age 65–74 years; CHA_2_DS_2_-VASc, congestive heart failure, hypertension, age ≥75 years, diabetes mellitus, prior stroke or transient ischaemic attack, vascular disease, age 65–74 years, and sex category; CHF, congestive heart failure; CKD, chronic kidney disease; COPD, chronic obstructive pulmonary disease; CTI, cavotricuspid isthmus; ESRD, end-stage renal disease; HLD, hyperlipidaemia; HTN, hypertension; IQR, interquartile range; LA, left atrial; LAAO, left atrial appendage occlusion; LVEF, left ventricular ejection fraction; OSA, obstructive sleep apnoea; PFA, pulsed-field ablation; PVI, pulmonary vein isolation; PWI, posterior wall isolation; SVC, superior vena cava; T2DM, type 2 diabetes mellitus; TIA, transient ischaemic attack.

A total of 379 patients received at least 1 follow-up with mean follow-up duration of 120.7 ± 138.6 days and 34.5% of patient remained on AAD at last follow-up. Using Kaplan–Meier survival analyses for the entire cohort, overall freedom from AF/AFL recurrence was 77.8% at 6 months, and 74.5% at 9 months for the entire cohort (*Figure 1B*).

Although limited by its single-centre nature, this study is one of the largest post-market outcomes report for the VLCC in the US comprising of more than 500 patients with balanced gender distribution. Compared with the European VARIPURE results, patients in this cohort represent a more advanced AF population.^5^ In our cohort 50.9% of the patients had non-paroxysmal AF (vs. 36.8% in VARIPURE) with significantly higher BMI (31.1 ± 6.7 vs. 27.8 ± 4.9), CHA_2_DS_2_-VA score (2.9 ± 1.6 vs. 1.9 ± 1.4), and CHF prevalence (37.2% vs. 14.8%). As a result, more than 2/3 of patients received lesion sets beyond PVI using VLCC, including 67.7% PWI and 49.9% SVC ablation compared with 1/3 in both the European experience and the recent REAL-AF reports.^5,8^ This likely accounted for the small increase in total procedure time due to more non-PV trigger ablations compared with the VARIPURE cohort. Moreover, this is one of the only studies that report the safety and efficacy of concomitant AF ablation with LAAO using the VLCC. From limited follow-up, we estimated the freedom from AF/AFL was 74.5% at 270 days using a 60-day blanking period for this advanced AF cohort (paroxysmal 83.0% vs. non-paroxysmal 62.2%), comparable to modern randomized control trials such as the ADVENT and CAPLA studies for paroxysmal and persistent AF.^9,10^ Our results show that both standalone PFA AF ablation and concomitant AF ablation with LAAO, performed with routine extra-PV PFA lesion sets using the VLCC, are safe, feasible, and effective in a high-volume, real-world registry.

## Data Availability

Data available on reasonable request.
